# Local Treatment Pulmonary Cavities by Injection

**Published:** 1874-05

**Authors:** William Pepper


Local Treatment of Pulmonary Cavities by Injections.—*By William Pepper, M.D.*—The only point which can be said to be demonstrated by the above cases is the possibility of puncturing superficial pulmonary cavities and injecting into them small quantities of dilute solution of iodine. And when it is seen how simple is the operation, it can only be wondered at that it has not long since been attempted, and its value determined. In estimating its danger, it is to be borne in mind that in the majority of cases when this treatment, if found of real curative value, will be called for, there exist pleuritic adhesions which will prevent the development of pneumothorax, even if so minute a puncture of the pulmonary pleura as is needed would allow the escape of any noteworthy amount of air.

Again, in treating the lung-tissue in this way, we are quite free from any fear of ill consequences from admitting air to the cavity, since, of course, it is already filled with it. The puncture would generally be made at a point of the lung where there are no large vessels or bronchi, and in conditions of the lung-tissue where many of the blood-vessels are obliterated. In case of an injury to a very small vessel, further, it does not seem likely that any uncontrollable hemorrhage would ensue. Still, it is evident that the danger of hemorrhage, as well as the ability to control it by injections of astringents through the canula, are questions requiring further observations to settle. My own limited experience, and the general considerations I have suggested, lead me to regard the danger of serious hemorrhage as but slight, if the puncture be carefully performed.

If it be established that the operation may be performed with safety, it remains to be considered to what classes of cases it is most suitable ; in what manner it may best be performed ; what local applications can advantageously be employed by this novel method, and what curative results can be obtained by it. It is evident that it finds its most simple application in the treatment of superficial pulmonary cavities. We have here an ulcerated surface separated from the skin merely by the intercostal tissues, thickened pleura, and a layer of infiltrated and indurated lung-tissue. It is difficult to imagine any serious consequences which can follow the puncture of such a cavity with a delicate needle. In such cases of phthisis with vomicæ, moreover, all means of treatment hitherto adopted are proverbially ineffectual. To how great an extent this is dependent upon the constitutional nature of many forms of phthisis is well known. But, in the light of our more correct knowledge of the local origin of some cases of phthisis, it is probable that their incurable character depends in a great measure upon our inability to maintain the affected part at rest, and to bring in contact with the diseased surface suitable agents of sufficient strength to modify the morbid action. It is probable, therefore, that this mode of treatment will find one of its most successful fields of application in chronic non-tuberculous cavities in the lungs, in cases where the remaining lung-tissue has not become the seat of secondary tuberculous formations. It is of course uncertain how much curative action we may be able to exert in such cases by any local application made through a canula. In the only case I have reported where the treatment has been continued long enough to produce any decided action, it is unquestionable that a certain degree of positive improvement has occurred, both in general symptoms and local signs. And I am encouraged to hope that, with further experience, definite modes of treatment may be formulated which will prove of material benefit in this hopeless class of cases.

In regard to the mode of making the puncture, I have hitherto employed the finest (No. 1) of the needles accompanying Dieulafoy's aspirator, and have used it with the "previous vacuum" attached. For the first exploratory diagnostic puncture it is probably desirable to employ an aspirator, as it would also be if it were desired to empty such cavities before injecting them. But for the continuance of the treatment it will perhaps be quite as well to use a capillary canula, with trochar which can be withdrawn, so that a syringe can be fitted to the canula and the injection made. I have employed local anæsthetics by freezing, and have directed the patients to take a full breath and to hold it before the puncture was made. It will, however, be understood that my procedure is in all respects purely a tentative one thus far, and that, if this mode of treatment prove of value and worthy of pursuance, many improvements will undoubtedly be made.

The only fluid which I have as yet injected has been dilute lugol's solution (*m*iv to f℥i), of which from four to ten minims have been injected. The entire absence of signs of irritation makes me confident that a larger quantity could be introduced without injury. This substance appeared suitable for the cases in which I have thus far operated. It is probable that other solutions, astringent or antiseptic, may be found preferable in some cases. In cases of local consolidations, solutions of iodine might also be expected to prove most beneficial.

I design employing a dilute solution of Monsel's salt for injection in suitable cases of serious hæmoptysis.

The practical value of this mode of treating pulmonary diseases is as yet uncertain. But it has appeared to me that, considering the almost hopeless nature of some of these lesions, the proof that a puncture may be made into the lung-tissue and remedial agents brought into direct contact wlth the seat of disease without any serious danger calls for a patient trial of it.

